# Genomic, Transcriptomic, and Proteomic Analysis Provide Insights Into the Cold Adaptation Mechanism of the Obligate Psychrophilic Fungus *Mrakia psychrophila*

**DOI:** 10.1534/g3.116.033308

**Published:** 2016-09-14

**Authors:** Yao Su, Xianzhi Jiang, Wenping Wu, Manman Wang, M. Imran Hamid, Meichun Xiang, Xingzhong Liu

**Affiliations:** * State Key Laboratory of Mycology, Institute of Microbiology, Chinese Academy of Sciences, Chaoyang District, Beijing 100101, China; † Novozymes (China) Investment Co. Ltd., Beijing 100085, China

**Keywords:** comparative genomics, transcriptomics, proteomics, temperature adaptation, basidiomycetous yeast

## Abstract

*Mrakia psychrophila* is an obligate psychrophilic fungus. The cold adaptation mechanism of psychrophilic fungi remains unknown. Comparative genomics analysis indicated that *M. psychrophila* had a specific codon usage preference, especially for codons of Gly and Arg and its major facilitator superfamily (MFS) transporter gene family was expanded. Transcriptomic analysis revealed that genes involved in ribosome and energy metabolism were upregulated at 4°, while genes involved in unfolded protein binding, protein processing in the endoplasmic reticulum, proteasome, spliceosome, and mRNA surveillance were upregulated at 20°. In addition, genes related to unfolded protein binding were alternatively spliced. Consistent with other psychrophiles, desaturase and glycerol 3-phosphate dehydrogenase, which are involved in biosynthesis of unsaturated fatty acid and glycerol respectively, were upregulated at 4°. Cold adaptation of *M. psychrophila* is mediated by synthesizing unsaturated fatty acids to maintain membrane fluidity and accumulating glycerol as a cryoprotectant. The proteomic analysis indicated that the correlations between the dynamic patterns between transcript level changes and protein level changes for some pathways were positive at 4°, but negative at 20°. The death of *M. psychrophila* above 20° might be caused by an unfolded protein response.

Fungi are found in all kinds of cold habitats, including permafrost, glaciers, and polar regions. The cold habitats (≤5°) occupy about 75% of the Earth’s biosphere. Fungi are better adapted to cold habitats than bacteria in solid media (Panikov and Sizova 2007). Psychrophilic micro-organisms (psychrophiles) exhibit an optimal growth temperature of about 15°, a minimal growth temperature of ≤0°, and cannot grow above 20° (Arthur and Watson 1976; Morita 1975). Some psychrophilic fungi are animal or plant pathogens, such as *Pseudogymnoascus destructans*, which causes white-nose syndrome of bats (Zhang *et al.* 2013). Although many psychrophilic fungi have been isolated, little is known about their cold adaptation mechanisms.

The majority of studies of microbiological cold adaptation mechanisms have focused on the cold stress response of mesophilic model organisms, such as *Escherichia coli*, *Bacillus subtilis*, and *Saccharomyces cerevisiae*. *E. coli* and *B. subtilis* respond to cold stress by inhibiting general protein synthesis and inducing cold shock proteins after growth has stopped for several hours (Goldstein *et al.* 1990; Willimsky *et al.* 1992; Jones *et al.* 1987). Synthesis of unsaturated fatty acids, accumulation of compatible solutes, and an antioxidant response are considered important responses for cold adaptation (Zhang *et al.* 2003; Chattopadhyay 2006; Borges *et al.* 2002; Casanueva *et al.* 2010). Whether psychrophilic fungi possess these cold adaptation mechanisms is unknown.

Cold shock and cold adaptation are two different processes. Cold shock means micro-organisms transferred from a higher temperature (*e.g.*, 37°) to a lower temperature (*e.g.*, 10°) in a short time, while cold adaptation refers to a micro-organism that exists under a steady state for a long time at low temperatures. Transcriptome profiles vary significantly at different time points after cold shock (Langin *et al.* 1995). As eukaryotes, fungi are quite different from bacteria in terms of their cold adaptation mechanism. A more moderate cold shock response is induced in yeast than in bacteria, while a strong cold shock response was observed in yeast after transfer to a near-freezing temperature (Kandror *et al.* 2004; Al-Fageeh and Smales 2006). Condition-specific alternative splicing has been documented to play an important role in adaptation to environmental stresses (Linde *et al.* 2015). However, most studies have focused on how psychrophilic micro-organisms survive and grow under low temperatures; little attention has been paid to why psychrophilic micro-organism cannot survive above 20°.

*Mrakia psychrophila* is an obligate psychrophilic fungus and a basidiomycetous yeast (Cystofilobasidiales, Tremellomycetes, Basidiomycota) with an optimal growth temperature range of 12–15°, and cannot grow above 20° (Supplemental Material, Figure S1). *M. psychrophila* has been isolated from diverse cold habitats, including Alpine glaciers and Antarctic soil (Xin and Zhou 2007; Turchetti *et al.* 2013).

In this study, the genome of *M. psychrophila* was analyzed and compared with other psychrophilic, thermophilic, and mesophilic fungi (Table S1). An integrative analysis of transcriptomic and proteomic data of *M. psychrophila* at 4, 12, and 20° was performed. This is the first integrated study providing insights into the cold adaptation mechanism of psychrophilic fungi from genomic, transcriptomic, and proteomic perspectives.

## Materials and Methods

### M. psychrophila strains and growth conditions

*M. psychrophila* NN053900 was isolated from permafrost on the Qinghai-Tibet Plateau. *M. psychrophila* was cultured on potato dextrose agar (PDA) and maintained at 4, 12, and 20° on PDA for 1 month, respectively. For cold shock treatments, *M. psychrophila* cultured at 12° were transferred to 4°.

### Genome sequencing and annotation

The genome of *M. psychrophila* was sequenced using Illumina HiSequation 2000 apparatus. The data were assembled using release v0.19 of Idba (Peng *et al.* 2010). We used homology and ab-initio prediction to identify protein-coding genes. For the homology-based gene prediction, we aligned the National Center for Biotechnology Information (NCBI) nr protein database to the *M. psychrophila* genome using TBLASTN and Genewise (Birney *et al.* 2004). We used FGENESH (Solovyev *et al.* 2006), GENEID (Blanco *et al.* 2002), GeneMark (Ter-Hovhannisyan *et al.* 2008), and SNAP (Korf 2004) for the ab-initio prediction. Protein alignments and ab-initio gene predictions were combined to build a consensus gene set using EvidenceModeler (Haas *et al.* 2008). Repeat sequences were identified using RepeatMasker (http://www.repeatmasker.org). Protein domains were identified with InterProScan (Apweiler *et al.* 2000). The final gene set was mapped to KEGG pathways using KAAS (Moriya *et al.* 2007). GO annotations were retrieved from the results of InterProScan and Blast2GO (Conesa *et al.* 2005). Homology-independent GO annotations were also predicted using FFPred (Minneci *et al.* 2013). Transfer RNAs (tRNAs) were predicted using tRNAScan-SE (Lowe and Eddy 1997). Other genome sequences and annotations used in comparative genomic analysis were downloaded from JGI (http://genome.jgi.doe.gov).

### Synonymous codon usage

We analyzed the patterns of synonymous codon usage in 15 completely sequenced fungal genomes, including four psychrophiles, three thermophiles, and eight mesophiles. We combined all the genes of each fungal genome, and calculated the relative synonymous codon usage (Vogel *et al.* 2010) for each fungus. We then used correspondence analysis to characterize the pattern of codon usage and map the pattern on to the distribution of codons and species (Lynn *et al.* 2002). Correspondence analysis was carried out using the “ca” package of R.

### Identification of optimal codon

For each gene of *M. psychrophila*, we calculated Nc (effective number of codons) and Nc′ (Nc corrected for nucleotide content) using ENCprime (Novembre 2002). We then calculated the correlation between each codon and the overall codon bias (Nc and Nc′) of each gene. The codon in each codon family showing the strongest and significantly negative correlation with the Nc or Nc′ was defined as the optimal codon (Hershberg and Petrov 2009). A correlation was considered significant at a P value ≤0.05/*n*, where *n* is the number of codons in the codon family. Spearman correlations were calculated using the R statistical package.

### Orthology, phylogenetic, and protein family evolutionary analyses

We used OrthoMCL (Li *et al.* 2003) to analyze the orthogroups of the 15 selected fungal genomes. A total of 16,325 orthogroups were identified, in which 296 orthogroups were single-copy orthologous genes. Based on the single-copy orthologous genes, a phylogenetic tree was constructed using RAxML 7.2.8 with the PROTGAMMAILGF model and 100 rapid bootstrap replications (Stamatakis 2006). The divergence time was estimated with the program r8s v1.7 (Sanderson 2003) and the origin of the Ascomycota at 500–650 million years ago (Lücking *et al.* 2009) as the calibration point. Each orthogroup from OrthoMCL was defined as a protein family. Protein family expansions and contractions were analyzed using CAFE (De Bie *et al.* 2006).

### Identification of orphan genes

Protein sequences were aligned against the NCBI nr database and SwissProt databases. All proteins with a BLAST hit with an *E*-value ≤10^−3^ were eliminated. The remaining proteins were defined as encoded by orphan genes (Wissler *et al.* 2013).

### RNA extraction and sequencing

RNA was isolated using a RNeasy Plant Mini Kit (QIAGEN) from *M. psychrophila* cultured at three temperatures: 4, 12 and 20°, with three replications per temperature. Sequencing libraries were generated using a NEBNextUltra RNA Library Prep Kit for Illumina (NEB) following the manufacturer’s recommendations. The prepared libraries were sequenced on an Illumina HiSequation 2000 platform and 100-bp paired-end reads were generated.

### Reads alignment and differential gene expression

TopHat v2.1.0 (Kim *et al.* 2013) was used to align the reads against the *M. psychrophila* genome sequences. The expression levels of transcripts were measured by fragments per kilobase per million mapped fragments using the Cufflinks software v2.2.1 (Trapnell *et al.* 2010). Differential expression was estimated and tested with the MA-plot-based method with random sampling model in the DEGseq package of R (Wang *et al.* 2010) between *M. psychrophila* cultured at 4 and 12°, as well as 20 and 12°; cutoff of the P value was 0.001. The P value and FDR of each gene are listed in Table S2.

### Annotation of alternative splicing events

SpliceGrapher (Rogers *et al.* 2012) was used to annotate and visualize all alternative splicing (AS) events, including intron retention, skipped exons, alternative 5′ splice sites (5′ SSs), and alternative 3′ SSs. Only AS events detected in more than two replicates were identified as true AS events.

### Real-time quantitative RT-PCR

RNA was isolated from *M. psychrophila* at 0, 10, 30 min and 1, 2, 4, 6, and 8 hr after cold shock. The reverse transcription reactions were performed with a Fast Quant kit (TIANGEN) in a 20-µl reaction system. The RT-solution was diluted 10 times, and 1 µl of the solution was used as the template in the 20 µl reaction system with 2×THUNDERBIRD SYBR qPCR Mix (TOYOBO). The quantitative PCRs were performed in technical triplicate using the BioRad CFX96 Touch Read-Time PCR detection system. The primers used in quantitative qPCR are listed in Table S3.

### Protein extraction, digestion, and iTRAQ labeling

*M. psychrophila* cultured at 4, 12, and 20° were harvested and lysed in a buffer containing 8 M urea, 30 mM 4-(2-hydroxyethyl)-1-piperazineethanesulfonic acid, 1 mM phenylmethanesulfonylfluoride, 2 mM ethylenediaminetetraacetic acid, and 10 mM dithiothreitol. After centrifugation at 20,000 × *g* for 30 min, the supernatants were reduced and alkylated by 10 mM DTT and 55 mM iodoacetamide. The treated proteins were precipitated in 80% acetone at −20° for 3 hr. After centrifugation at 20,000 × *g* for 30 min, the precipitants were resuspended in 50% tetraethylammonium bromide and 0.1% sodium dodecyl sulfate and again centrifuged at 20,000 × *g* for 30 min. The protein concentrations were determined using the Bradford method. The proteins were digested with trypsin at 37° for 12 hr. The tryptic peptides were labeled using an iTRAQ reagents 8-plex multiplex kit (Applied Biosystems) following the manufacturer’s protocol.

### NanoLC-MS/MS analysis and protein identification

The iTRAQ labeled peptide mixtures were separated on a Luna SCX strong cation exchange high performance liquid chromatography column (Phenomenex) and desalted with strata-X C18 column (Phenomenex). The mobile phase A was 0.1% formic acid in water, while mobile phase B was 0.1% formic acid in acetonitrile. Peptides were eluted in a linear gradient of 5–80% mobile phase B over 65 min. The elutes were directly entered into a Thermo Fisher Q Exactive mass spectrometer, setting in positive ion mode and data-dependent manner with full MS scan from 350 to 2000 m/z, resolution at 70,000. The MS/MS data were searched using Mascot 2.3.01 (Matrix Science, Boston, MA) against the database with all predicted proteins in *M. psychrophila*. Only proteins identified below the 1% global FDR were qualified for further quantitative data analysis. The fold changes in protein abundance were defined as the median ratio of all significantly matched spectra with tag signals. Differentially expressed proteins (DEPs) were identified with fold changes >1.5. When analyzing correlation between the transcriptome and the proteome, transcript level is qualified with FPKM; transcript level change and protein level change are qualified with fold change of DEG and DEP, respectively.

### Data availability

The genome sequence of *M. psychrophila* was submitted to NCBI (Bioproject PID PRJNA304674). The transcriptome data have been deposited in the NCBI Gene Expression Omnibus and are accessible through GEO Series accession number GSE76769. The proteomics data were deposited at PRIDE Archive under accession number PXD004881. Reference numbers for data available in public repositories: Genome: Bioproject PID PRJNA304674 (NCBI), Transcriptome: GSE76769 (GEO), Proteome: PXD004881 (PRIDE Archive).

## Results

### Genome sequencing and annotation

The draft sequence of *M. psychrophila*’s genome was obtained using the whole genome shotgun Sanger sequencing approach to ∼400× coverage. The genome was assembled into 1975 contigs, with a total size of 27.8 Mb (Table 1). Comparison with two phylogenetically related basidiomycetous yeasts showed that its genome size is larger than *Cryptococcus neoformans* (19.1 Mb), but smaller than *Dioszegia cryoxerica* (39.5 Mb). The overall GC content is 53.8%, which is higher than *C. neoformans* (48.5%), but lower than *D. cryoxerica* (56.1%). 76.4% of the predicted gene models (4579) in *M. psychrophila* had matches in the GenBank nonredundant protein database (BLASTP; *E*-value <1e−5) and about 21.3% of the predicted gene models (1276) were identified as orphan genes. The GC content of the coding sequences in the psychrophilic fungi *M. psychrophila* (56.5%) and *D. cryoxerica* (58.6%) is higher than that in the mesophilic fungus *C. neoformans* (51.2%). GC content in the third codon position (GC3) in even higher in *M. psychrophila* (63.3%) and *D. cryoxerica* (68.3%) than that in *C. neoformans* (52.1%). The average number of exons in *M. psychrophila* is 8.0, which is higher than that of *C. neoformans* (6.5) and *D. cryoxerica* (5.4).

**Table 1:** Genomic features of *M. psychrophila*

Characteristic	*M. psychrophila*
Total contigs length (Mb)	27.8
Number of contigs	1976
Contig N50 (kb)	33.7
Repeat sequences (%)	5
Percentage of CDS (%)	36.6
GC (%) genome	53.8
GC (%) CDS	56.5
GC3 (%)	63.3
Protein-coding gene number	5994
Mean exon number per gene	8
Mean gene length (bp)	1697.6
Mean protein length (amino acid)	564.9

CDS, coding sequence; GC3, GC content at the third codon position.

### Synonymous codon usage bias

**Figure 1:**
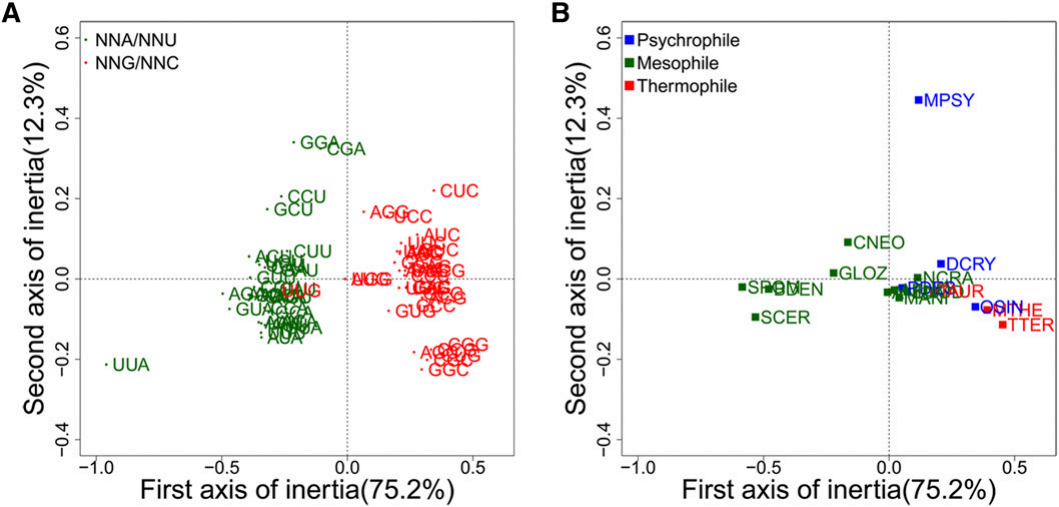
Correspondence analysis of the relative synonymous codon usage from 15 fungal genomes. (A) The distribution of synonymous codons along the first and second axes of the correspondence analysis. Codons ending in A or U are shown in green and codons ending in G or C are shown in red. (B) The distribution of species along the first and second axes of the correspondence analysis. Psychrophiles are shown in blue, mesophiles are shown in green, and thermophiles are shown in red.

The genes of each genome were combined for correspondence analysis of relative synonymous codon usage. Codons were separated along the horizontal axis by their GC3 values. All the psychrophiles and thermophiles showed a preference for codons ending in G and C, while some mesophiles prefer to use codons ending in A and U, such as *C. neoformans* and *S. cerevisiae*. *M. psychrophila* is separated from other fungi along the vertical axis because of its preferential usage of codons GGA and CGA for Gly and Arg, respectively (Figure 1), which was also supported from the optimal codons analysis (Table S4). Gly and Arg account for 7.6 and 6.0% of the amino acid composition in *M. psychrophila*, respectively. The copy numbers of tRNAs corresponding to GGA or CGA in *M. psychrophila* are higher than those in other basidiomycetous yeasts, which are in turn higher than the copy numbers of tRNAs corresponding to other synonymous codons of Gly or Arg in *M. psychrophila* (Table S5).

### Lineage-specific expansion and contraction of gene families

**Figure 2:**
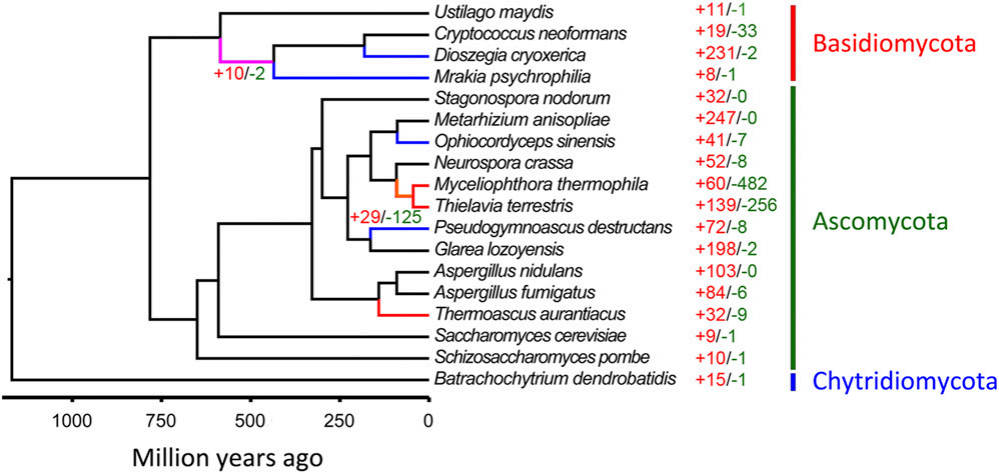
Gene family expansion and contraction in fungal genomes. The gene families that have undergone expansion and contraction are shown in red and green, respectively. The psychrophilic fungi branches are shown in blue; the thermophilic fungi branches are shown in red; the Tremellomycetes branch is shown in purple; and Chaetomiaceae branch is shown in orange.

Annotated genes were organized into 16,325 families based on orthoMCL, in which 3221 families contain genes from *M. psychrophila*. We identified eight gene families that are expanded in *M. psychrophila* compared with other fungi, while one gene family has been lost in *M. psychrophila*. Two gene families are significantly expanded that do not have orthologs in other fungi and are predicted to be homologs encoding receptor binding, ATP binding, and regulation of gene expression. Other expanded gene families are related to “glucosidase activity” (GO:0015926), “peptidase activity” (GO:0008233), and “transmembrane transport” (GO:0055085). The gene family lost from *M. psychrophila* is an orphan gene family of *D. cryoxerica*. The *Tremellomycetes* clade (Figure 2) comprises two psychrophilic fungi (*M. psychrophila* and *D. cryoxerica*) and *C. neoformans*, while the genus *Cryptococcus* is also frequently isolated from cold habitats (Thomas-Hall and Watson 2002). There are 10 expanded gene families and two contracted gene families in the *Tremellomycetes* clade. The expanded gene families in the *Tremellomycetes* clade are related to transport (GO:0022857, “transmembrane transporter activity”) and the two-component system (GO:0004673, “protein histidine kinase activity”; GO:0000155, “two-component sensor activity”; GO:0000156, “two-component response regulator activity”). One expanded gene family in *M. psychrophila* and three expanded gene families in the *Tremellomycetes* clade all belong to the major facilitator superfamily (MFS). There are 83 genes that belong to MFS in *M. psychrophila*. The MFS is a large and ancient family of secondary transporters that are energized by electrochemical proton motive force (Sá-Correia *et al.* 2009). MFS proteins are involved in antifungal resistance and nutrition transport (Gaur *et al.* 2008). The gene family related to glycoside hydrolase has been expanded in two psychrophilic fungi, *M. psychrophila* and *D. cryoxerica*, while the gene family containing an oligopeptide transporter (OPT) domain has been expanded in two psychrophilic fungi *M. psychrophila* and *Ophiocordyceps sinensis* (Xiao *et al.* 2013). Genes with an OPT domain are responsible for transporting of oligopeptides (Perlin *et al.* 2014).

### Differentially expressed genes (DEGs) at 4 and 20°

**Figure 3:**
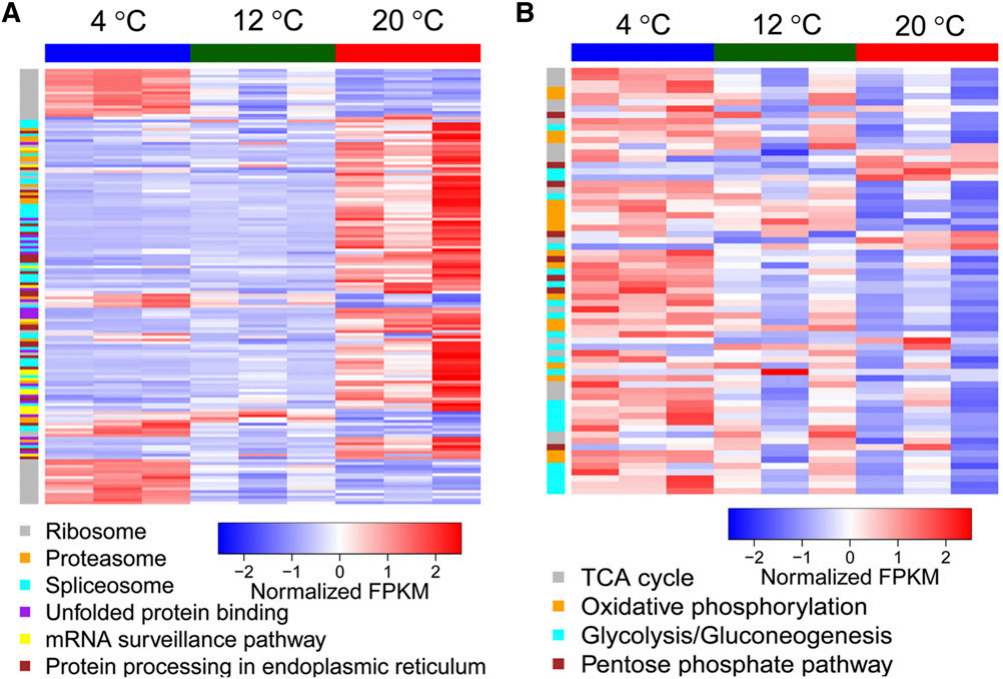
(A) Heatmap of differentially expressed genes (DEGs) involved in transcription, post-transcriptional regulation, translation, and post-translational regulation. (B) Heatmap of DEGs involved in energy metabolism.

Compared with the optimal temperature for growth at 12°, 684 genes were upregulated and 844 genes were downregulated at 4°, while 1202 genes were upregulated and 1078 genes were downregulated at 20°. Significant differences in transcriptome profiles were observed between *M. psychrophila* cultured at 4 and 20°. For DEGs at 4°, GO terms related to the ribosome, translation, and transporter are enriched, suggesting that translation may play an important role at near-freezing temperatures (Table 2). For DEGs at 20°, GO terms related to ribosomes are also enriched, which implied that translation is also important for survival at 20° (Table 3). By performing KEGG pathway analyses, DEGs at 4° were identified as being involved in the ribosome, glycolysis/gluconeogenesis, pentose phosphate, tricarboxylic acid (TCA) cycle, and fatty acid metabolism pathways. For DEGs at 20°, the ribosome, spliceosome, and oxidative phosphorylation pathways were significantly enriched. Ribosomal genes were upregulated at 4° and downregulated at 20°. Proteins involved in the proteasome, spliceosome, unfolded protein binding, and mRNA surveillance were upregulated at 20° (Figure 3A). Energy metabolism was upregulated at near-freezing temperature (Figure 3B). Desaturase *DesC* (MPSY2164), which is involved in the biosynthesis of unsaturated fatty acids, was only upregulated at 4°, while another desaturase *Fad2* (MPSY3203) was upregulated at 4 and 20°. Nine genes of the steroid biosynthesis pathway were upregulated at 4°, but none of them was upregulated at 20° (Figure S2). Glycerol 3-phosphate dehydrogenase (*Gpd*, MPSY651), the rate-controlling enzyme of glycerol formation, was upregulated at 4°, which indicated that glycerol serves as a compatible solute in *M. psychrophila*. Trehalose-6-phosphate synthase (*Tps*, MPSY304), the enzyme for trehalose synthesis, was not upregulated at 4 or 20°, which suggested that there is no trehalose accumulation in *M. psychrophila*. Although reactive oxygen species (ROS) are a threat at low temperatures, superoxide dismutase (*Sod*, MPSY659), which is involved in catalase involving antioxidant defense, was not upregulated at 4 or 20°. *Ire1* (MPSY2892) and *Kar2* (MPSY3525), which are involved in the unfolded protein response (UPR), were both upregulated at 20°. In the 133 MFS genes of *M. psychrophila*, 39 genes were upregulated and 13 genes were downregulated at 4°, while nine genes were upregulated and 48 genes were downregulated at 20°. Of 1276 orphan genes of *M. psychrophila*, 469 orphan genes (36.8%) were differentially expressed.

**Table 2:** GO enrichment analysis of DEGs at 4° in *M. psychrophila*

GO ID	GO Term	GO Class	P value	Adjusted P value
GO:0010467	Gene expression	BP	5.42E−15	3.78E−12
GO:0016491	Oxidoreductase activity	MF	6.71E−14	4.67E−11
GO:0008152	Metabolic process	BP	1.48E−12	1.03E−09
GO:0030529	Ribonucleoprotein complex	CC	6.52E−11	4.53E−08
GO:0055114	Oxidation-reduction process	BP	9.71E−11	6.73E−08
GO:0003735	Structural constituent of ribosome	MF	2.90E−10	2.01E−07
GO:0005840	Ribosome	CC	2.39E−09	1.65E−06
GO:0006412	Translation	BP	8.86E−06	6.11E−03
GO:0005975	Carbohydrate metabolic process	BP	9.39E−06	6.47E−03
GO:0022857	Transmembrane transporter activity	MF	2.00E−05	1.37E−02
GO:0043169	Cation binding	MF	2.80E−05	1.93E−02
GO:0006007	Glucose catabolic process	BP	4.43E−05	3.04E−02

The significance of the enrichment was calculated using Fisher’s exact test following a conservative correction for multiple tests (false discovery rate <0.05, Holm’s correction). BP, biological process; CC, component; DEGs, differentially expressed genes; MF, molecular function.

**Table 3:** GO enrichment analysis of DEGs at 20° in *M. psychrophila*

GO ID	GO Term	GO Class	P value	Adjusted P value
GO:0003735	Structural constituent of ribosome	MF	3.88E−10	3.56E−07
GO:0005840	Ribosome	CC	7.05E−09	6.45E−06
GO:0010467	Gene expression	BP	6.43E−08	5.87E−05
GO:0016491	Oxidoreductase activity	MF	4.16E−07	3.79E−04
GO:0008152	Metabolic process	BP	4.38E−06	3.99E−03
GO:0030529	Ribonucleoprotein complex	CC	6.83E−06	6.22E−03

The significance of the enrichment was calculated using Fisher’s exact test following a conservative correction for multiple tests (false discovery rate <0.05, Holm’s correction). BP, biological process; CC, component; DEGs, differentially expressed genes; MF, molecular function.

### Alternative splicing at different temperatures

**Figure 4:**
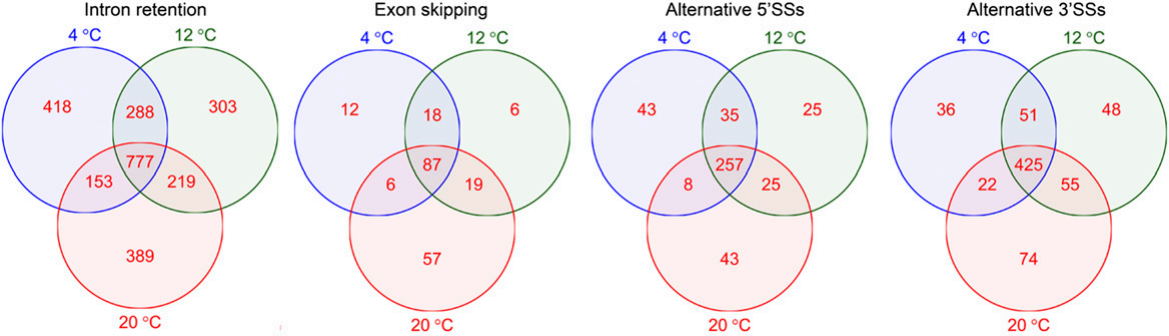
Venn diagram showing unique and shared genes that undergo alternative splicing between and among *M. psychrophila* at 4, 12, and 20°.

**Figure 5:**
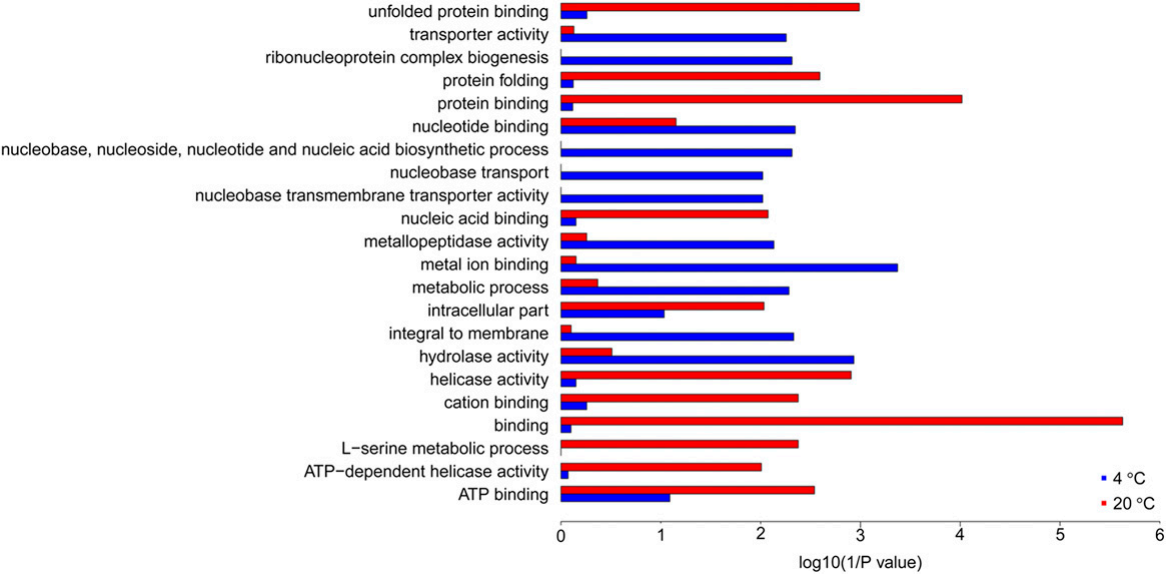
Enrichment of GO terms in genes that undergo intron retention at 4 and 20°.

Alternative splicing is prevalent in *M. psychrophila*. We identified 14,481 AS events including 8910 intron retention, 663 exon skipping, 1756 alternative 5′ SSs, and 2968 alternative 3′ SSs. All the predicted splicing sites were restricted to the canonical GT-AG or GC-AG pattern. Intron retention (IR) was the most prevalent AS event at 4, 12, and 20°; alternative 3′ SSs were the second most common AS events (Table 4). About one third of all the genes in *M. psychrophila* had undergone AS and three quarters of the alternatively spliced genes contained retained introns (Table 5). IR in most genes leads to premature termination of translation. Multiple occurrences of AS events were observed in >400 genes in *M. psychrophila*, so the total AS events (Table 4) are higher than those detected in genes (Table 5). There was a significant difference in AS between *M. psychrophila* at different temperatures. Some genes only underwent AS at a certain temperature; for example, IR in 418 genes only occurred at 4° (Figure 4). IR was the most prevalent form of AS in *M. psychrophila*; therefore, we analyzed the functional categories of genes that underwent IR only at 4 or 20°. Genes that underwent IR at 4° are related to “transporter activity” (GO:0005215), “ribonucleoprotein complex biogenesis” (GO:0022613), “metal ion binding” (GO:0046872), and “hydrolase activity” (GO:0016787). Genes that underwent IR only at 20° are related to “unfolded protein binding” (GO:0051082), “helicase activity” (GO:0004386), and “L-serine metabolic process” (GO:0006563) (Figure 5). Pathway analysis also indicated that IR tends to occur in specific pathways at 4 or 20°. For example, seven genes involved in the spliceosome undergo IR at 20°, while only one gene underwent IR at 4° in that pathway.

**Table 4:** Alternative splicing events at different temperatures

Temperature (°)	Intron Retention	Exon Skipping	Alternative 5′SS	Alternative 3′SS	Total
4	1581 (43.8%)	168 (4.6%)	733 (20.3%)	1150 (31.8%)	3613
12	1431 (40.0%)	180 (5.0%)	749 (20.9%)	1242 (34.7%)	3576
20	1319 (38.2%)	221 (6.4%)	703 (20.3%)	1234 (35.7%)	3455

SS, splice site.

**Table 5:** Genes that undergo alternative splicing at different temperatures

Temperature (°)	Intron Retention	Exon Skipping	Alternative 5′SS	Alternative 3′SS	Total
4	1636 (77.8%)	123 (5.8%)	343 (16.3%)	534 (25.4%)	2104
12	1587 (75.9%)	130 (6.2%)	342 (16.3%)	579 (27.7%)	2092
20	1538 (74.8%)	169 (8.2%)	333 (16.2%)	576 (28.0%)	2056

SS, splice site.

### Cold stress response at near-freeze temperature

**Figure 6:**
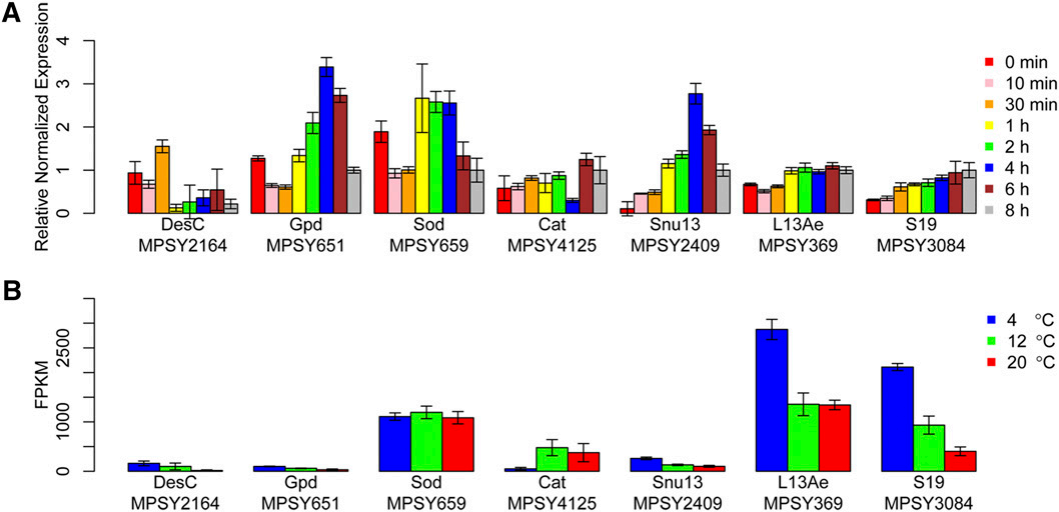
(A) Gene expressions analyzed by qPCR of selected genes at eight time points after cold shock in *M. psychrophila*. (B) Gene expressions analyzed by RNA-seq of selected genes at three temperatures after cold adaptation in *M. psychrophila*. The bars represent the SD calculated from three technical replicates of each experiment.

To analyze the transcriptome profile in response to cold stress, we monitored the expression levels of seven genes at 0, 10, and 30 min and 1, 2, 4, 6, and 8 hr after cold shock (from 12 to 4°) via quantitative real-time PCR (qPCR) (Figure 6A). The desaturase gene *DesC* (MPSY2164), which was upregulated at 4°, was downregulated at 10 min and upregulated at 30 min, after which it was expressed at a low level from 1 to 8 hr. *Gpd* (MPSY651), a core gene for the biosynthesis of glycerol, was downregulated immediately after cold shock, but upregulated from 1 hr. *Sod* (MPSY659) was also downregulated immediately after cold shock and upregulated from 1 hr, which indicated that *Sod* (MPSY659) is important for ROS reduction after cold shock. Catalase (*Cat*, MPSY4125), which degrades hydrogen peroxide, was expressed at low level after cold shock. The gene encoding small nuclear ribonucleoprotein-associated protein 1 (*Snu13*, MPSY2409), which is involved in the spliceosome, was upregulated before 4 hr and downregulated after 4 hr. Ribosomal genes were upregulated at 4°, the expression levels of ribosomal genes *L13Ae* (MPSY369) and *S19* (MPSY3084) were examined and shown to increase gradually after cold shock. The qPCR result showed that gene expression levels fluctuate after cold shock. When compared with RNA-seq analysis of cold adaptation (Figure 6B), ribosomal genes *L13Ae* (MPSY369) and *S19* (MPSY3084) show the same tendency.

### Differentially expressed proteins at different temperature

Isobaric tag for relative and absolute quantitation (iTRAQ)-based proteomics analysis was performed to obtain the protein expression profiles in *M. psychrophila* cultured at 4, 12, and 20°. Overall, 12,107 unique peptides, corresponding to 1673 proteins, were identified. Considering FDR <0.01 and unique peptide numbers ≥2, 1345 nonredundant proteins were obtained, of which 41 are encoded by orphan genes. Compared with *M. psychrophila* cultured at 12°, there were 54 DEPs (27 upregulated and 27 downregulated) and 75 DEPs (28 DEPs upregulated and 47 downregulated) at 4 and 20° respectively. Comparing the DEPs at 4 and 20°, eight DEPs were upregulated, while 10 DEPs were downregulated at both temperatures. At 4°, two proteins of the TCA cycle (citrate synthase MPSY4093 and LSC2, MPSY4552), one protein of glycolysis (PDC, MPSY2287), and one ribosomal protein (RPSB, MPSY5379) were downregulated, while one protein of amino acid metabolism (GLNA, MPSY123) and one MFS transporter (MPSY2821) were upregulated. At 20°, four proteins of the TCA cycle including two citrate synthase (MPSY4127 and MPSY4093), DLST(MPSY3759) and LSC2 (MPSY4552), one protein of oxidative phosphorylation (MTCP1, MPSY4350), and one protein of glycerophospholipid metabolism (CLD1, MPSY5022) were downregulated, while one heat shock protein (DNAJ, MPSY1312) and two MFS transporters (MPSY4181 and MPSY2821) were upregulated. The results indicated that energy metabolism is involved in the adaptation of *M. psychrophila* at 4 and 20°, and the heat shock response might be induced at 20°.

### Correlation between the transcriptome and the proteome

**Figure 7:**
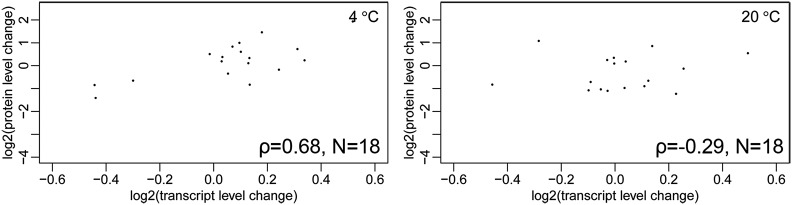
Correlation ρ between protein level change and transcript level change for genes involved in glycerophospholipid metabolism. N, gene number in the pathway.

The transcriptome and proteome of *M. psychrophil*a were compared: 12 and 8 genes are upregulated at both mRNA and protein levels at 4 and 20°, while 19 and 21 genes are downregulated at both mRNA and protein levels at 4 and 20°. The mRNA abundance and the expression change of proteins in *M. psychrophila* were compared between 4 and 20°. The relationship between the transcript level and the protein level change was positive (ρ = 0.09, P = 1.7 × 10^−3^, Figure S3A) at 4°, but significantly negative (ρ = −0.11, P = 8.3 × 10^−5^, Figure S3B) at 20°. This indicated that proteins encoded by highly expressed transcripts tend to be upregulated at 4°, but downregulated at 20°. Protein level changes and corresponding transcript level changes at 4 and 20° were compared and the overall correlation coefficients were 0.25 and 0.09 at 4 and 20°, respectively. Furthermore, the correlation of dynamic patterns between protein level changes and transcript level changes by each pathway were highly variable between the two temperatures (Table S6), for example, the change of transcript and protein levels in glycerophospholipid metabolism were significantly positively correlated at 4° (ρ = 0.68, P = 1.84 × 10^−3^), but not correlated (ρ = −0.29, P = 0.24) at 20° (Figure 7). Glycerophospholipid metabolism is important for membrane fluidity. The same tendencies have been observed for fatty acid metabolism, glycolysis/gluconeogenesis, and RNA degradation.

## Discussion

Comparative genomic, transcriptomic, and proteomic analyses have revealed the cold adaptation mechanisms in *M. psychrophila*. Although some strategies for cold adaptation by *M. psychrophila* are shared with other psychrophiles, some new findings that are different from other psychrophiles were observed. Codon usage bias and AS might contribute to the cold adaptation of *M. psychrophila*. In addition, no growth above 20° could be caused, at least partially by accumulation of unfolded proteins in the endoplasmic reticulum (ER).

The transcriptome profile fluctuated after cold stress; therefore, cold adaptation of psychrophiles cannot be simply explained by the cold stress response of mesophilic model organisms. Cold adaptation and cold shock are distinct processes. Cold adaptation means that micro-organisms are cultured in a steady state at a low temperature for a long time, whereas in cold shock, the culture temperature is downshifted suddenly. Energy metabolism is repressed during the cold stress response of bacteria *Pseudomonas putida* (Frank *et al.* 2011), but it is upregulated in cold adapted *M. psychrophila*. Cold shock induces accumulation of glycogen and trehalose, as well as upregulation of *Sod* and *Cat*, in psychrotolerant fungi *Penicillium olsonii* (Gocheva *et al.* 2009). Although glycerol is thought to be accumulated, activation of the antioxidant response was not observed in *M. psychrophila* cultured at near-freeze temperature. Similarly, in *S. cerevisiae*, trehalose did not serve as a compatible solute in the steady state at low temperature (Tai *et al.* 2007), but accumulation of trehalose was observed in response to cold shock at near-freezing temperatures (temperature downshift to near-freeze temperature) (Kandror *et al.* 2004). Genes involved in RNA polymerase and rRNA processing, cytosolic ribosomal proteins, and the general stress response were upregulated at the early, middle, and late phases of cold stress response of *S. cerevisiae* respectively (Sahara *et al.* 2002). The time-dependent transcriptomic variation may reflect the transition from cold shock to cold adaptation.

*M. psychrophila* produces unsaturated fatty acids (UFAs) to maintain membrane fluidity and accumulates glycerol as a compatible solute at 4°, indicated by upregulation of *DesC* (P value = 4.17E−9) and *Gpd* (P value = 2.21E−6). The MFS transporter and energy metabolism are also important for cold adaptation. Membrane fluidity is essential for organisms to survive in psychrophilic habitats and the desaturase *DesC* is generally upregulated to produce unsaturated fatty acids as observed in *B. subtilis* after cold shock (Aguilar *et al.* 1998). Desaturase is a key enzyme for biosynthesis of UFAs, which are beneficial for keeping membrane fluidity at low temperatures. *M. psychrophila* is predicted to accumulate glycerol as a compatible solute by upregulation of *Gpd* at 4°, which is responsible for synthesis of glycerol. *S. cerevisiae* also accumulates glycerol for freeze protection in an HOG-dependent manner (Butler *et al.* 2009). Ribosomes serve as sensors of heat and cold shock in *E. coli* (VanBogelen and Neidhardt 1990). Ribosomal genes or ribosome-biogenesis genes are upregulated in *M. psychrophila* and *S. cerevisiae* respectively (Tai *et al.* 2007). The translation process may slow down at low temperature, and more ribosomes might be needed for translation.

Specific codon usage bias might also be a benefit for the cold adaptation of *M. psychrophila*, especially GGA for Gly and CGA for Arg. There are several reasons for the codon usage bias in *M. psychrophila*. First, codon usage bias might influence mRNA folding structure (Trotta 2013) and special codon usage may facilitate translation at low temperatures. Second, codon usage bias may have coevolved with the copy number of the corresponding tRNAs. The speed and accuracy of translation might be influenced by tRNA abundance (Higgs and Ran 2008; Shah and Gilchrist 2010). In fact, copy numbers of tRNA corresponding to GGA and CGA are the highest among those corresponding to other codons encoding Gly and Arg. Thus, the specific codon usage bias in *M. psychrophila* might be beneficial for the speed and accuracy of translation at low temperatures. Third, codon usage bias might be caused by genetic code ambiguity. In *Candida albicans*, Leu codon CUG can be translated to Ser (97%) or Leu (3%) (Zhou *et al.* 2013). There may be such genetic code ambiguity in *M. psychrophila*; therefore, it must use rare codons as optimal codons to avoid nonsynonymous mutations. The MFS gene family is expanded in *M. psychrophila* and other species in the *Tremellomycetes* clade, which may contribute to the accumulation of nutrients from the environment (Perlin *et al.* 2014). MFS transporters are also important for acid tolerance of *Penicillium funiculosum* (Xu *et al.* 2014). Energy metabolism is upregulated in *M. psychrophila* at 4°, as well as in *Candida glabrata* under acidic stress (Zhou *et al.* 2011). *M. psychrophila* may need more ATP for transport, biosynthesis of UFAs, and glycerol at 4°.

The nonsurvival of *M. psychrophila* above 20° might be owing to the induction of UPR. Genes involved in unfolded protein binding, protein processing in the ER, and proteasome, spliceosome and mRNA surveillance are upregulated at 20°. Upregulation of genes related to unfolded protein binding and genes involved in UPR indicated that there was an accumulation of unfolded proteins at 20°. Unfolded proteins accumulating in the ER has been named as ER stress (Gardner *et al.* 2013). UPR is triggered to maintain protein-folding homeostasis of the ER and induce expression of genes involved in protein-folding, vesicle trafficking, and protein degradation (Hou *et al.* 2014; Labunskyy *et al.* 2014). The protein processing in the endoplasmic reticulum pathway is upregulated to enhance the protein-folding capacity of the ER. Upregulation of the proteasome indicated that unfolded proteins are degraded in a ubiquitin-mediated way. In addition to post-translational regulation, mRNA expression may be downregulated by spliceosome-mediated decay (SMD) given that the spliceosome pathway is upregulated at 20°. In SMD, splicing mutants are produced by the spliceosome and degraded by mRNA surveillance systems (Volanakis *et al.* 2013). The negative correlation between protein level change and transcript level change might reflect ubiquitin-mediated protein degradation and SMD. If a cell cannot rebuild homeostasis, UPR will become cytotoxic and lead to apoptosis (Gardner *et al.* 2013). AS also plays an important role in the cold adaptation of *M. psychrophila*. Genes related to unfolded protein binding are especially alternatively spliced and upregulated at 20°. IR, the most frequent form of AS, is observed in plants, fungi, and mammals (Ner-Gaon *et al.* 2004; Braunschweig *et al.* 2014; Sebé-Pedrós *et al.* 2013). AS could enhance proteome diversity; most IR events produce premature termination codon transcripts (Kwon *et al.* 2014), which are turned over by nonsense-mediated decay (NMD) (de Lima Morais and Harrison 2010). Cold-induced AS is also observed in the alga *Chlamydomonas reinhardtii* (Valledor *et al.* 2013); thus AS might be an important adaptive mechanism for eukaryotic micro-organisms.

**Figure 8:**
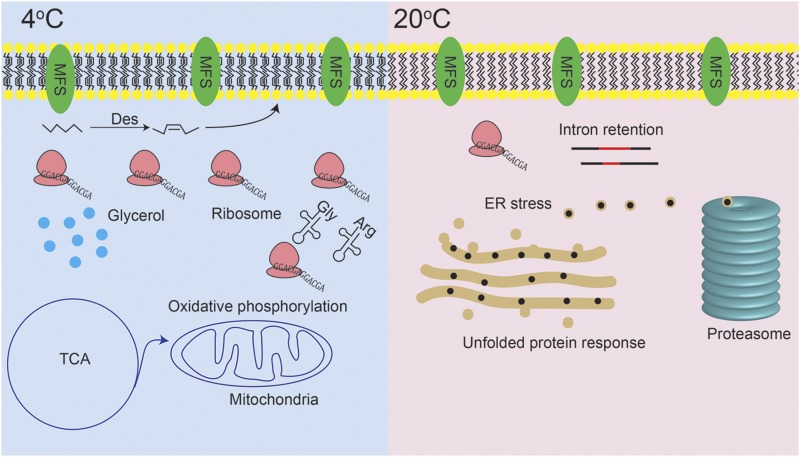
Global view of the adaptation mechanism of *M. psychrophila* at 4 and 20°.

This study provided a comprehensive analysis of the cold adaptation mechanism in the obligate psychrophilic fungus *M. psychrophila* using omics approaches. *M. psychrophila* shares some cold adaptive strategies with other micro-organisms, including desaturation of fatty acids, accumulation of the compatible solute glycerol, upregulation of ribosome-associated proteins, and energy metabolism. There are special traits of *M. psychrophila* that are not observed in other psychrophiles. The codon usage bias of *M. psychrophila* is very different from other psychrophilic, mesophilic, and thermophilic fungi. Thousands of genes are alternatively spliced in *M. psychrophila* and IR is the most common form. Codon usage bias and AS may contribute to the cold adaptation of *M. psychrophila*. In addition to adaptation at low temperatures, we also tried to explain the upper temperature limit for growth. Above 20°, unfolded proteins accumulate in the ER. The overloaded ER triggers the UPR, which upregulates genes related to protein folding and protein degradation. If the UPR cannot rescue ER homeostasis, apoptosis is induced. Although the results of the cold stress response in the model organism are heuristic, cold adaptation in the steady state is different from cold shock, and psychrophiles adopt more flexible strategies to cope with cold. Our results provide a global view of the cold adaptation mechanism of psychrophilic fungi (Figure 8).

## 

## Supplementary Material

Supplemental Material
