# Supplementary material for: Genomic, Transcriptomic, and Proteomic Analysis Provide Insights Into the Cold Adaptation Mechanism of the Obligate Psychrophilic Fungus Mrakia psychrophila
Source: G3 (Bethesda). 2016 Sep 14;6(11):3603–13. doi: 10.1534/g3.116.033308 (PMC5100859; doi:10.1534/g3.116.033308)
Supplement: Supplemental Material [file supp_6_11_3603__index.html]

Genomic, Transcriptomic, and Proteomic Analysis Provide Insights Into the Cold Adaptation Mechanism of the Obligate Psychrophilic Fungus Mrakia psychrophila — Supplemental Material 

# Genomic, Transcriptomic, and Proteomic Analysis Provide Insights Into the Cold Adaptation Mechanism of the Obligate Psychrophilic Fungus *Mrakia psychrophila*

## Supplemental Material for Su *et al.*, 2016

**Files in this Data Supplement:**

- Table S1 - Selected fungal genomes for comparative genomics analysis. (.xls, 23 KB)
- Table S2 - P value and FDR of DEGs. (.xls, 867 KB)
- Table S3 - Primers used for qPCR. (.xls, 23 KB)
- Table S4 - Optimal codons identified in fungi. (.xls, 24 KB)
- Table S5 - Copy number of tRNAs corresponding to Gly and Arg in basidiomycetous yeasts. (.xls, 21 KB)
- Table S6 - Correlation between transcript level changes and protein level changes of each pathway. (.xls, 45 KB)
